# ‘Where is my gap’: mechanisms underpinning PARP inhibitor sensitivity in cancer

**DOI:** 10.1042/BST20241633

**Published:** 2025-02-10

**Authors:** Lauryn Buckley-Benbow, Alessandro Agnarelli, Roberto Bellelli

**Affiliations:** Centre for Cancer Cell and Molecular Biology, Barts Cancer Institute, Queen Mary University of London, Charterhouse Square, Barbican, London EC1M 6BQ, U.K.

**Keywords:** BRCA, PARPi, POLE3-POLE4, replicative gaps

## Abstract

The introduction of poly-ADP ribose polymerase (PARP) inhibitors (PARPi) has completely changed the treatment landscape of breast cancer susceptibility 1–2 (BRCA1–BRCA2)-mutant cancers and generated a new avenue of research in the fields of DNA damage response and cancer therapy. Despite this, primary and secondary resistances to PARPi have become a challenge in the clinic, and novel therapies are urgently needed to address this problem. After two decades of research, a unifying model explaining sensitivity of cancer cells to PARPi is still missing. Here, we review the current knowledge in the field and the increasing evidence pointing to a crucial role for replicative gaps in mediating sensitization to PARPi in BRCA-mutant and ‘wild-type’ cancer cells. Finally, we discuss the challenges to be addressed to further improve the utilization of PARPi and tackle the emergence of resistance in the clinical context.

## Introduction: BRCAs and PARPs

Genomic instability is a hallmark of cancer and understanding its nature has provided avenues for patient-tailored therapies. The paradigmatic example of this concept is the synthetic lethality between loss of breast cancer susceptibility 1–2 (BRCA1-BRCA2) in cancer and poly-ADP ribose polymerase (PARP) inhibitors (PARPi) [1]. The concept behind this phenomenon has inspired a new wave of research in the field of the DNA damage response (DDR) and cancer therapeutics, leading to the introduction in clinical trials of several agents targeting the DDR [2]. BRCA1 and BRCA2 are the most frequently mutated genes in hereditary breast and ovarian cancer syndromes; loss of BRCA1 and BRCA2 is indeed associated with a lifelong increased incidence of breast and ovarian cancer of ~80% and ~25–50%, respectively [3]. BRCA1-2 are mostly known for their role in homologous recombination (HR), a type of DNA double-strand break (DSB) repair, which requires RAD51-mediated strand exchange for sister chromatid-dependent error free repair [4]; HR is strictly regulated in a cell cycle-dependent manner and antagonized by the 53BP1-SHIELDIN pathway, which prevents DSB resection and promotes nonhomologous end joining, an alternative error-prone DSB repair pathway [5]. Importantly, in addition to its classic role in HR, RAD51 and BRCA1-2 have a crucial role in protecting stalled replication forks from promiscuous nuclease activities and preventing accumulation of ssDNA gaps at the replication fork [6,7]. The interplay between the loss of these functions in genome stability and sensitization to PARPi in BRCA-mutant cancers is not completely defined.

**Figure 1:**
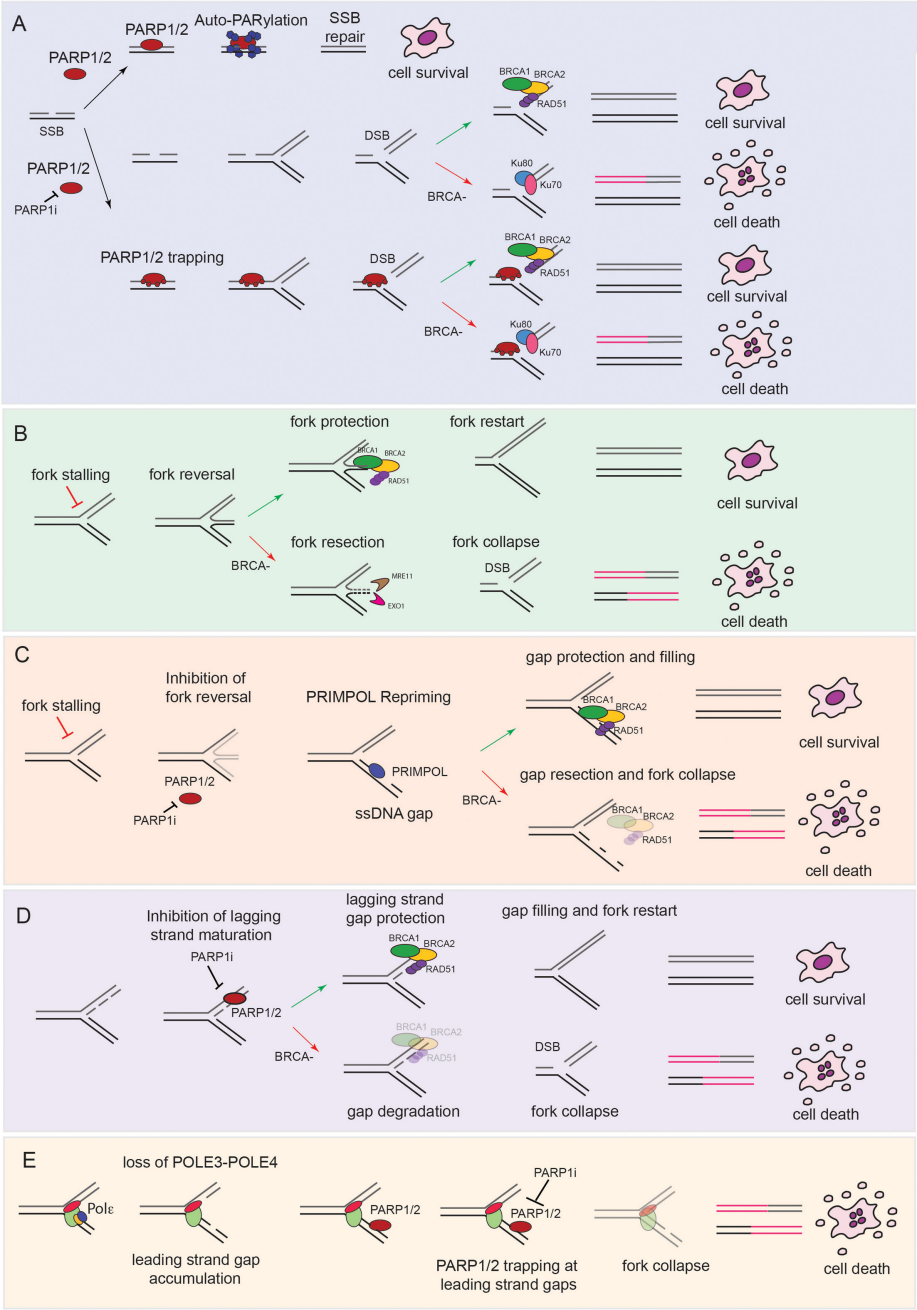
Mechanisms of sensitization to PARPi. **(A)** Representative scheme of the classic ‘SSB to DSB’ model of sensitization to PARPi: in the lower panel, PARP is presented as ‘trapped’ on ssDNA upon treatment with PARPi. **(B)** Representative scheme of the ‘fork reversal/deprotection’ model showing fork degradation upon reversal in BRCA-mutant cells. **(C)** Representative scheme showing how inhibition of fork reversal and PRIMPOL-dependent repriming leads to ssDNA gap accumulation/deprotection in BRCA-null cells. **(D)** Representative scheme of the classic ‘ssDNA gap**’** model showing how defective lagging strand maturation upon PARPi treatment leads to lagging strand gaps and toxicity in BRCA-mutant cells. **(E)** Representative model showing how loss of POLE3-POLE4 unleashes leading strand gap accumulation upon treatment with PARPi. Abbreviations: BRCA, breast cancer susceptibility; DSB, double-strand break; PARPi, poly-ADP ribose polymerase inhibitors; SSB, single-strand break.

PARPs are a class of enzymes catalyzing the addition, in a NAD^+^-dependent manner, of poly-(ADP-ribose) (PAR) chains on substrate proteins, a process known as PARylation, and themselves (autoPARylation) [1] (Figure 1). The main targets of clinically relevant PARPi are PARP1 and PARP2, with the latter being most likely associated with PARPi-mediated hematologic toxicities [1,8]. PARP1 has been mostly characterized as a single-strand break (SSB) sensor in base excision repair (BER); more specifically, PARP1 binds to SSBs via its N-terminal zinc finger domains; this leads to a conformational change and the release of an autoinhibitory interaction between the helical and the catalytic ADP-ribosyl transferase domains, which promotes NAD+ access to the catalytic site. The subsequent PARylation of specific proteins induces chromatin relaxation and increased access of DNA repair proteins to DNA damage sites to promote their repair [1,9]. PARylation is particularly important for the recruitment of XRCC1 in BER [9]; this, in turn, promotes downstream recruitment of Ligase III and Polβ for gap filling and ligation [9]. PARP1 also PARylates itself to promote its own chromatin release, an event required for effective resolution of DNA repair reactions [1,9]. AutoPARylation of PARP1 is also negatively regulated by PAR glycohydrolase (PARG) and ADP-ribosylhydrolase 3 (AHR3), with important implications for DNA repair and sensitization to PARPi [10]. Finally, in addition to its well-studied role in BER, PARP1 is activated at DSBs and stalled replication forks to promote their stabilization and restart [11]. Commercially available PARP1-2 inhibitors are nicotinamide mimicking compounds that bind the catalytic domain of PARP1-2 and prevent PARylation. The PARPi currently approved in the clinic also prolongs PARP1-2 retention at sites of DNA damage, a phenomenon also known as ‘trapping’, which prevents resolution of DNA repair reactions [1].

### An updated framework of cancer cell sensitivity to PARPi

In 2005, two landmark manuscripts published in Nature described for the first time the extreme sensitivity of BRCA-mutant cells to PARPi [12,13]. These findings have been followed by the rapid introduction of PARPi in clinical trials and the current approval for the treatment of HR-deficient ovarian, breast, pancreatic, and prostatic cancers of Olaparib, Talazoparib, Rucaparib, and Niraparib [14–20]. Given the well-known roles of BRCA1-2 in DSB repair by HR and the established function of PARP1-2 in SSB repair, the initial framework to explain the synthetic lethality between mutations of BRCA1-2 and PARPi pointed to unrepaired SSBs, upon inhibition of PARP1-2, causing replication-coupled DSBs; the latter would normally require HR for effective repair. Loss of HR, in BRCA-mutant cells, would lead to alternative and catastrophic end joining events, increasing chromosomal instability and leading to cell death (‘SSB to DSB model’; Figure 1A). An initial challenge to this simplified view was the finding that retention of PARP1-2 at DNA lesions, or trapping, is crucial to explain sensitivity to PARPi [21] (Figure 1A). Consistently with this, the trapping potency of clinically available PARPi correlates with their efficacy [21]. Furthermore, mutation or loss of PARP1 can lead to resistance to PARPi *in vitro* [22].

Importantly, to complicate the SSB to DSB model, BRCA proteins have a crucial role in replication fork protection (Figure 1B). Replication fork stalling is associated with regression of replication forks into a four-way structure, a process known as fork reversal, which is crucial to stabilize replication forks and allow their restart [23]. Work from several laboratories has shown that BRCA1-2 are specifically required to protect reversed replication forks from MRE11- and EXO1-dependent degradation [24–27]. The absence of this protective mechanism causes excessive fork resection and collapse (‘fork reversal/deprotection model’; Figure 1B). Of note, resistance to PARPi has been associated with restoration of fork protection in both BRCA1- and BRCA2-mutant backgrounds, suggesting a relevant role in mediating PARPi sensitivity [28,29]. However, although fork deprotection remains a well-defined feature of BRCA-mutant cells, fork protection experiments are performed in the presence of hydroxyurea, which induces fork slowing/stalling and does not recapitulate the events associated with PARPi treatment in cells. PARP1 has indeed a crucial role in fork reversal; hence, the treatment with PARPi prevents fork reversal in cells [30,31] (Figure 1C). In further support of this notion, Maya-Mendoza et al. have shown that incubation with PARPi leads to replication fork acceleration and not slowing [32].

The reconciliation between these findings came from the discovery that the treatment with PARPi unleashes post-replicative ssDNA gaps accumulation in BRCA1-null cells, a feature strongly associated with sensitization to these compounds [33] (‘ssDNA gap model’; Figure 1D). The origin of ssDNA gap accumulation upon PARPi treatment is not completely defined, and multiple sources likely contribute to this phenomenon, including PRIMPOL-dependent repriming and impeded maturation of lagging strands. First, PARP inhibition, as well as inhibition of fork reversal by knockdown of SMARCAL1, is known to stimulate PRIMPOL-mediated repriming, leading to ssDNA gap accumulation, which requires RAD18-dependent translesion synthesis (TLS) or template switch for repair [34,35]. Consistently with this, RAD18 and the TLS polymerase, Polζ, are required for viability of BRCA-mutant cells [36]. More recently, the accumulation of ssDNA gaps has been linked to defective lagging strand maturation upon PARPi treatment [33,37]. PARP1 acts as a sensor of unligated Okazaki fragments; thus, treatment with PARPi impedes Okazaki fragments maturation [37,38]. In agreement with a lagging strand mechanism in BRCA-null cells, work in *Xanopus laevis* egg extracts has shown an important role for POLθ in preventing lagging strand gap accumulation upon depletion of RAD51 and BRCAs [39]. Similarly, inhibition of POLθ by small molecule inhibitors induced post-replicative ssDNA gaps in BRCA-mutant cells, suggesting a direct role for POLθ in filling post-replicative lagging strand gaps [40]. Interestingly, recent work from Hanthi et al. pointed to abasic site protection by RAD51 being crucial in preventing replicative gap accumulation [41]; this finding might help explain the previously described role of the SMUG1 glycosylase in ssDNA gap accumulation in BRCA-null cells [36]. Whatever mechanism might initially cause replicative gap formation, BRCA-deficient cells are defective in protecting ssDNA gaps from MRE11-dependent degradation [35]; these data can reconcile the replication fork protection and ssDNA gap mechanisms of PARPi sensitization; furthermore, ‘trapped’ ssDNA gaps are likely to persist into mitosis and the following S phase potentially leading to DSBs and ‘classic HR-dependent’ sensitization to PARPi [42]; this would finally reconcile the ‘SSB to DSB’ vs. ‘ssDNA gap’ hypothesis. Of note, while increasing evidence points to ssDNA gaps as the initial DNA lesion that sensitizes cancer cells to PARPi, work from the Jasin laboratory has recently suggested that, at least in a BRCA2-mutant setting, loss of HR is the ‘ultimate’ cause of sensitization to PARPi [43]. It is likely that the contribution of ssDNA gaps vs. HR might vary depending on the genetic background and experimental setting. As such, a complete and definitive separation of function might be difficult to achieve.

### A leading strand model for PARPi sensitization

While lagging strand gaps are likely to play a prominent role in a BRCA-mutant context, we and others have challenged the view that lagging strand gaps are the ‘ultimate sensitizers’ to PARPi [44,45]. Indeed, we have recently shown that the loss of the POLE3-POLE4 subunits of DNA polymerase epsilon (Polε), the leading strand DNA polymerase, leads to a striking increased sensitivity to PARPi (Figure 1E). Loss of POLE3-POLE4 was not associated with the evidence of HR deficiency, as visualized by RAD51 foci formation, and sensitivity to methyl methanesulfonate or mytomycin C [44–46]. On the contrary, POLE3-POLE4 KO cells rapidly accumulated into the G2 phase of the cell cycle upon PARPi treatment; this was associated with increased chromatin levels and phosphorylation on S33 of replication protein A, a classic marker of replication stress and ssDNA accumulation. Furthermore, S1 nuclease experiments pointed to the accumulation of PRIMPOL-dependent post-replicative gaps in this condition. Of note, ssDNA gaps were specifically observed upon treatment with PARPi but not in the unchallenged condition. We envisaged two non-mutually exclusive possibilities to explain this phenomenon: (i) replicative gaps might ‘normally’ form in POLE3-POLE4 KO cells and be resolved in a PARP1/2-dependent manner; PARP inhibition would lead to persistent unrepaired/trapped ssDNA gaps and replication catastrophe or conversion into DSBs into the subsequent cell cycle; it is worth noting that POLE3-POLE4 KO cells were not sensitive to transient silencing of PARP1 and PARP2 by siRNAs, while the knockdown of PARP1-2 rescued sensitization to PARPi, suggesting a crucial role for PARP1-2 trapping in this context. (ii) Transient fork reversal would normally allow POLE3-POLE4 KO cells to sustain their replicative defect and permit re-engagement of Polε with its DNA substrate and/or the switch to Polδ or a TLS polymerase; inhibition of fork reversal by PARPi would lead to excessive PRIMPOL-dependent repriming and replicative gap accumulation. Consistently with this possibility, POLE3-POLE4 KO cells were sensitive to silencing of SMARCAL1, a crucial factor involved in fork reversal, and RAD18, which is required for Proliferating Cell Nuclear Antigen (PCNA) ubiquitylation and TLS polymerase recruitment. A combination of (i) and (ii) is also possible. Interestingly, the loss of BRCA1 did not lead to replicative gap accumulation in POLE3-POLE4 KO cells in untreated conditions, which suggests a specific role for BRCA1-2 in protecting lagging strand gaps. Consistently with this, silencing of BRCA1 further sensitized POLE3-POLE4 KO cells to PARPi, suggesting a non-epistatic mechanism of sensitization to these compounds. Finally, and consistently with a HR-independent mechanism of sensitization to PARPi, loss of 53BP1 was not associated with a rescue of PARPi sensitivity in POLE3-POLE4 KO cells. While suggesting that targeting Polε might represent a therapeutic opportunity to tackle resistance to PARPi, these data indicate that replicative gap accumulation upon PARPi treatment might represent the initial event that drives sensitization to PARPi in a BRCA-mutant and wild-type setting.

### Transcription–replication conflicts and PARPi sensitivity

In addition to its well-known role in DNA repair, PARP1 and PARP2 have also been implicated in transcriptional regulation, RNA processing, and ribosome biogenesis [47,48]; as such, enzymatic inhibition of PARPs is likely to affect different biological processes [48]. Recently, work from Petropoulos et al. has also suggested that PARP1, in concert with the replisome factors TIMELESS and TIPIN, is required to resolve transcription–replication conflicts (TRCs) [49]. Consistently with this, PARPi treatment induces accumulation of TRCs and pathologic R-loops; thus, loss of BRCAs, which have been previously implicated in prevention and resolution of TRC and R-loops, might result in synthetic lethality with enzymatic inhibition of PARPs [49–51].

### Mechanisms of resistance to PARPi

**Figure 2:**
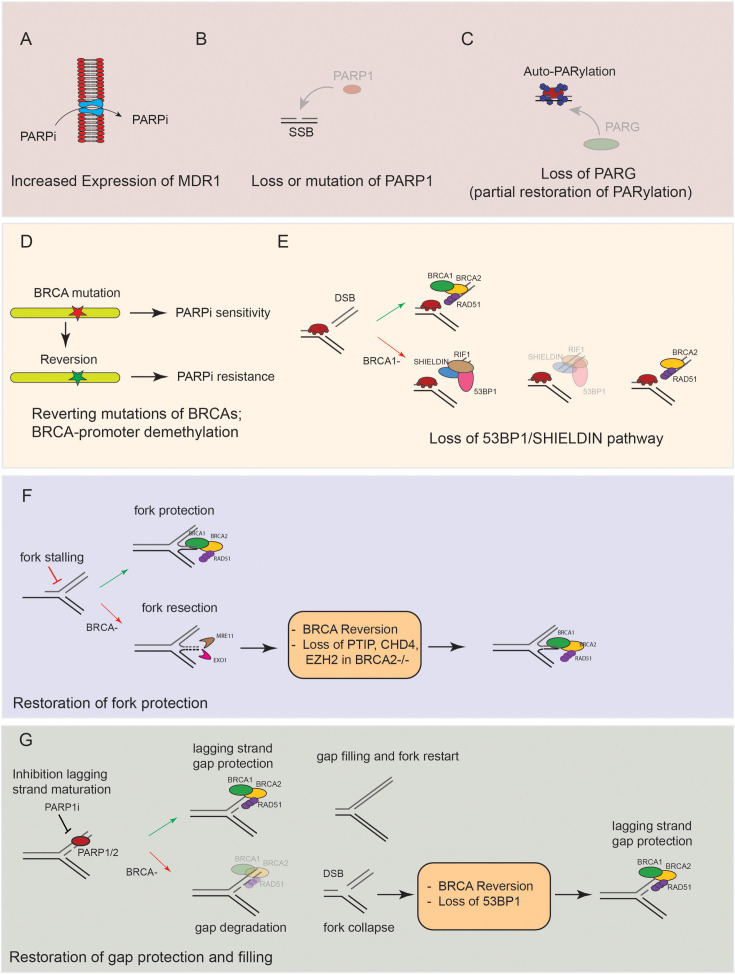
Mechanisms of resistance to PARPi. **(A)** Representative scheme showing how MDR1 up-regulation leads to increased cellular efflux and resistance to PARPi. **(B)** Representative scheme showing how loss or mutation of PARP1 reduces sensitivity to PARPi by preventing PARP trapping. **(C)** Representative scheme showing how loss of PARG expression leads to a partial restoration of PARylation and reduced PARP trapping. **(D)** Representative scheme of BRCA mutations and their reversion in cases of PARP inhibitor resistance; demethylation of BRCA promoters is also included in this category of resistance mechanisms. **(E)** Representative scheme showing how loss of 53BP1/SHIELDIN leads to restoration of resection and PARP inhibitor resistance in BRCA-null cancers. **(F)** Representative scheme showing how revertant BRCA mutations and loss of PTIP, CHD4, and EZH2 lead to restoration of fork protection and resistance to PARPi. **(G)** Representative scheme showing how revertant BRCA mutations and loss of 53BP1 restore gap protection/filling and PARPi sensitivity. Abbreviations: BRCA, breast cancer susceptibility; MDR, multidrug resistance; PARPi, poly-ADP ribose polymerase inhibitor.

Primary and secondary (or acquired) resistance to PARPi is a challenge in the clinic, particularly in non-ovarian HR-deficient cancers, where PARPi have shown more limited efficacy. Several mechanisms of PARPi resistance have been identified *in vitro* (e.g. cancer cell lines and patient-derived organoids) and *in vivo* (clinical samples) with significant differences based on genetic background (e.g. BRCA1 vs. BRCA2 mutant) and tumor types (e.g. breast vs. ovarian cancer). The mechanisms of resistance to PARPi can be broadly classified as ‘restoring’ or not BRCA/HR, with the latter being mostly characterized *in vitro* (Figure 2). One of the first mechanisms of resistance identified is up-regulation of the multidrug resistance 1 (MDR1) transporter, initially reported in a genetically engineered BRCA1 mouse model [52](Figure 2A) and later in tissues from PARPi-treated cancers [53]. In addition to this, both mutations and loss of PARP1 have been detected in PARPi-resistant cancer cells [28] (Figure 2B). Similarly, loss of PARG has been identified as a mechanism of resistance to PARPi, initially in a BRCA2-mutant model [54]. (Figure 2C). The mechanistic explanation for this phenomenon is that the loss of PARG expression allows minimal PARylation events to take place upon PARPi treatment; these include PARP1-2 autoPARylation, which allows PARP1-2 dissociation from DNA, thus reducing its trapping (Figure 2C). The most frequent and clinically relevant mechanism of resistance to PARPi is, however, constituted by ‘revertant’ mutations in BRCA genes, which restore HR, as well as replication fork/ssDNA gap protection (Figure 2D, F and G). In other words, PARPi sensitivity due to BRCA mutations can be ‘simply’ reverted by a secondary mutation in the BRCA gene that restores its function. Initially identified *in vitro* in 2008, this mechanism of resistance has been documented in the majority of cases *in vivo* and in different stages and tumor types (breast, ovarian, and prostatic cancer) [55–59] (Figure 2D). Importantly, reversions have not only been identified in BRCA1-2-mutant carrier but also in PALB2-, RAD51C-, and RAD51D-mutant cancers pointing to a general mechanism of PARPi resistance [60,61]. In addition to this, since HR defects can be caused by BRCA genes promoter methylation, a phenomenon well known for BRCA1 and RAD51C, an alternative mechanism of HR restoration is the loss of promoter hyper-methylation. This event has been detected in both patient-derived organoids and patient samples, using biopsies of ovarian cancers before and after platinum progression [62,63]. Finally, an alternative mechanism of restoration of HR, particularly relevant in a BRCA1-mutant context, is caused by the loss of anti-resection factors of the 53BP1-RIF1-SHIELDIN pathway [64–67]. This phenomenon, well characterized *in vitro*, has recently emerged also in the clinical setting in breast cancer patients [57,59]. Mechanistically, the specific role of replication fork stability and ssDNA gap accumulation in mediating resistance to PARPi remains difficult to ascertain given the high frequency of revertant mutations in BRCA proteins. Despite this, *in vitro* resistance to PARPi in BRCA1- and BRCA2-mutant cells is associated with restoration of fork protection and loss of ssDNA gap accumulation [28,29] (Figure 2E–2F). Consistently with this, in BRCA2-deficient cells, the loss of the MLL3/4 complex protein PTIP was specifically associated with the regain of fork protection and resistance to PARPi [29]. A similar effect has been described upon the loss of the nucleosome remodeling factor CHD4 and the methyltransferase EZH2[68,69]. Of note, the loss of 53BP1 was also associated with the restoration of gap protection in BRCA1-null cells [33].

### Hampering or preventing the emergence of resistance to PARPi

Resistance to targeted therapies is unfortunately common in cancer and a significant clinical issue for PARPi. However, given the relatively safe profile of PARPi and their extensive use in the maintenance setting, combination therapies that can prevent or tackle the emergence of resistance have been extensively investigated (Table 1). A combination strategy in advanced clinical studies is based on the use of ATR inhibitors. Previous research has indeed shown that the combination of Olaparib and Ceralasertib, a commonly used ATR inhibitor (ATRi), can resensitize PARPi- and platinum-resistant ovarian cancers to PARPi [28,70]. Consistently with this, promising phase II results of the CAPRI trial have shown ~50% overall response rate with the ATRi+PARPi combination in patients who had progressed after PARPi treatment [71]; a similar approach has been investigated with CHK1 and WEE1 inhibitors, despite higher toxicities might limit these combinations [72,73]. Alternative promising targets for combinatorial DDR therapies are USP1, POLζ, and POLθ. USP1 is a de-ubiquitylating enzyme that targets mono-ubiquitinated PCNA and prevents TLS polymerase recruitment at the replication fork. The loss of USP1 is synthetic lethal with BRCA1[74]; mechanistically, BRCA1-mutant cells accumulate ssDNA gaps in the absence of USP1 and undergo replication catastrophe [75]. Consistently with this, USP1 inhibitors have showed promising *in vitro* and *in vivo* activities against BRCA-mutant cells and cancer models, target PARPi resistance, and are currently being investigated in phase I trials [75,76]. Similarly, BRCA-mutant cells rely on POLζ for gap filling and viability and, as such, JH-RE-06, a small molecule inhibitor of REV1-POLζ, reduced *in vitro* and *in vivo* growth of BRCA-mutant cancer cells [36]. In addition to this, HR-deficient cancer cells are dependent on POLθ-dependent microhomology-mediated end joining (MMEJ) and gap filling for viability [39,40,77,78]. Thus, a small molecule inhibitor of POLθ sensitizes BRCA-null cells to PARPi and tackles resistance to PARPi in multiple BRCA-mutant models, comprising 53BP1-SHIELDIN-null cells [79]. Interestingly, sequence analyses of reversion events identified in the clinic have highlighted a critical role for POLθ-dependent MMEJ in this process [80]. As such, the combination of PARP and POLθ inhibitors might tackle emergence of resistance in the first place and is currently being exploited in phase I–II trials. Finally, since BER intermediates are the main substrates for PARP trapping, an alternative approach is increasing the accumulation of such intermediates to further sensitize BRCA-mutant cells to PARPi. Such avenues might involve inhibition of the BER chromatin remodeling factor ALC1, as well as the nucleotide sanitizers DNPH1 or ITPA [81–83].

**Table 1:** Therapeutic approaches to target PARPi resistance in cancer.

Mechanism of sensitization to PARPi	Mechanism of resistance to PARPi	Therapeutic approach	Target and stage of development
Defective HR-dependent DSB repair	BRCA reversions	Preventing the establishment of resistance with combination therapies (POLθi, ATR/CHK1i, DNPH1i/ALC1i)	POLθi (Phase I–II) ATRi (Phase I–II–III) CHK1i (Phase I–II) DNPH1, ALC1 (pre-clinical)
	Loss of 53BP1-SHIELDIN pathway	Targeting POLθ or the ATR/CHK1 axis	POLθi (Phase I–II) ATRi (Phase I–II–III) CHK1 (Phase I–II)
Defective replication fork protection	Restoration of fork protection (BRCA reversion, loss of PTIP, CDH4, EZH2)	Targeting the ATR/CHK1 axis	ATRi (Phase I–II–III) CHK1 (Phase I–II)
Replication gap accumulation	Restoration of gap filling/repair (BRCA reversion, loss of 53BP1)	Targeting POLθ, ATR/CHK1, REV1, or USP1 to increase gap accumulation and/or prevent their filling/repair	POLθi (Phase I–II) ATRi (Phase I–II–III) CHK1i (Phase I–II) USP1 (Phase I) REV1i (pre-clinical)

Table summarizing the mechanisms of sensitization and resistance to PARPi together with the potential therapeutic approaches to tackle the emergence of resistance and the specific targets and stage of clinical development.

BRCA, breast cancer susceptibility. DSB, double-strand break. HR, homologous recombination. PARPi, poly-ADP ribose polymerase inhibitors.

In addition to ‘DDR-focused’ combinations, preclinical data have supported combinatorial PARPi and immunotherapies. Mechanistically, PARPi treatment induces accumulation of DSBs and cGAS-dependent STING activation, which promotes CD8+T cell recruitment and cancer cell killing [84,85]. Furthermore, treatment with PARPi has been directly associated with up-regulation of PDL-1 expression which has supported combination therapies with antibodies targeting PD-1/PDL-1[86]. With this in mind, Olaparib and Niraparib are currently undergoing clinical evaluation in combination with Durvalumab and Pembrolizumab, respectively [87,88]. Furthermore, in ovarian cancer, research has supported addition of anti-angiogenic therapies to PARPi [89,90]. Consistently with this, the combination of Olaparib or Niraparib with Bevacizumab, an anti-VEGF agent, was associated with improved progression-free survival; these findings led to the approval of these combinations as a first-line maintenance after platinum-based therapy in ovarian cancer patients [91,92]. Finally, in prostatic cancers, preclinical evidence has pointed to a ‘BRCAness’ phenotype induced by androgen receptor (AR) signaling inhibition [93,94]. As such, abiraterone, a novel anti-AR agent, and enzalutamide, a classic AR inhibitor, have been employed in trials for the treatment of metastatic castration resistance prostatic cancer in combination with Olaparib or Talazoparib; the overall positive results of these studies led to the recent approval of these combinations, irrespective of HR status [95,96].

In summary, understanding the mechanisms that sensitize cancer cells to PARPi has provided new therapeutic opportunities to tackle resistance to these compounds, extend their utilization in the clinic, and open new avenues for cancer therapeutics. Dissecting the role of replicative gaps as ‘entry’ point for ‘BRCAness’ holds promise to further increase their effective utilization in the clinic in the fight against cancer.

**Perspectives**

- The introduction of poly-ADP ribose polymerase inhibitors (PARPi) has re-shaped the treatment landscape of breast cancer susceptibility syndrome-mutant cancers.
- Replicative gaps recently emerged as a crucial determinant of sensitivity to PARPi in cancer.
- Novel combinatorial approaches are required to tackle the emergence of resistance to PARPi in the clinic.

## Abbreviations

BER: base excision repair
BRCA: breast cancer susceptibility
DRR: DNA damage response
DSB: double-strand break
HR: homologous recombination
MDR1: multidrug resistance 1
PARPi: poly-ADP ribose polymerase inhibitors
SSB: single-strand break
TLS: translesion synthesis
TRCs: transcription–replication conflicts
